# The type III secretion system effector EspO of enterohaemorrhagic *Escherichia coli* inhibits apoptosis through an interaction with HAX-1

**DOI:** 10.1111/cmi.13366

**Published:** 2021-06-24

**Authors:** Sharanya Chatterjee, Sujinna Lekmeechai, Nicolas Constantinou, Ewa A. Grzybowska, Zuzanna Kozik, Jyoti S. Choudhary, Cedric N. Berger, Gad Frankel, Abigail Clements

**Affiliations:** 1 Department of Life Sciences, MRC Centre for Molecular Bacteriology and Infection, Imperial College, London, UK; 2 Department of Molecular and Translational Oncology, Maria Sklodowska-Curie National Institute of Oncology, Warsaw, Poland; 3 Functional Proteomics Group, The Institute for Cancer Research, London, UK

**Keywords:** infection, mechanism of action, microbial-cell interaction, virulence

## Abstract

Many enteric pathogens employ a type III secretion system (T3SS) to translocate effector proteins directly into the host cell cytoplasm, where they subvert signalling pathways of the intestinal epithelium. Here, we report that the anti-apoptotic regulator HS1-associated protein X1 (HAX-1) is an interaction partner of the T3SS effectors EspO of enterohaemorrhagic *Escherichia coli* (EHEC) and *Citrobacter rodentium*, OspE of *Shigella flexneri* and Osp1**_STYM_** of *Salmonella enterica* serovar Typhimurium. EspO, OspE and Osp1_STYM_ have previously been reported to interact with the focal adhesions protein integrin linked kinase (ILK). We found that EspO localizes both to the focal adhesions (ILK localisation) and mitochondria (HAX-1 localisation), and that increased expression of HAX-1 leads to enhanced mitochondrial localisation of EspO. Ectopic expression of EspO, OspE and Osp1_STYM_ protects cells from apoptosis induced by staurosporine and tunicamycin. Depleting cells of HAX-1 indicates that the anti-apoptotic activity of EspO is HAX-1 dependent. Both HAX-1 and ILK were further confirmed as EspO1-interacting proteins during infection using T3SS-delivered EspO1. Using cell detachment as a proxy for cell death we confirmed that T3SS-delivered EspO1 could inhibit cell death induced during EPEC infection, to a similar extent as the anti-apoptotic effector NleH, or treatment with the pan caspase inhibitor z-VAD. In contrast, in cells lacking HAX-1, EspO1 was no longer able to protect against cell detachment, while NleH1 and z-VAD maintained their protective activity. Therefore, during both infection and ectopic expression EspO protects cells from cell death by interacting with HAX-1. These results suggest that despite the differences between EHEC, C. *rodentium, Shigella* and S. *typhimurium* infections, hijacking HAX-1 anti-apoptotic signalling is a common strategy to maintain the viability of infected cells.

## 1 Introduction

Shiga toxigenic *Escherichia coli* (STEC, aka VTEC) strains, particularly those that carry the locus of enterocyte effacement (LEE) pathogenicity island and belong to serotype O157:H7 (enterohaemorrhagic E. coli—EHEC), cause diarrhoea, haemorrhagic colitis and haemolytic uremic syndrome (HUS). The elaboration of Shiga toxins (Stx) is responsible for HUS, which is the leading cause of acute paediatric renal failure (Kaper, Nataro, & Mobley, 2004). In 2018, there were 8,314 confirmed cases of STEC reported in the European Union, including 411 cases of HUS, 64.3% of which were in children aged 0–4 years (European Food Safety, European Centre for Disease,, & Control, 2019). No vaccines or specific treatments, other than rehydration and nutritional supplementation, are currently available to deal with these infections (reviewed in Goldwater & Bettelheim, 2012). EHEC strains colonise the human and animal intestinal mucosa via attaching/effacing (A/E) lesions (reviewed in Frankel et al., 1998). This infection strategy is shared by two other A/E pathogens, entero-pathogenic *E*. *coli* (EPEC), which is an important cause of infant diarrhoea in low-income countries (reviewed in (Chen & Frankel, 2005; Garmendia, Frankel, & Crepin, 2005) and the related mouse pathogen *Citrobacter rodentium* (CR; reviewed in Mundy, MacDonald, Dougan, Frankel, & Wiles, 2005; Collins et al., 2014). The genes required for A/E lesion formation are carried on pathogenicity islands and mobile genetic elements (Iguchi et al., 2009; McDaniel, Jarvis, Donnenberg, & Kaper, 1995; Tobe et al., 2006), including the LEE, which encodes transcriptional regulators, chaperones, a type III secretion system (T3SS; responsible for effector protein translocation), the adhesin intimin and six effector proteins including Tir that serves as an intimin receptor in infected cells (reviewed in Wong et al., 2011). T3SS effectors of A/E pathogens target diverse signalling pathways, including host cell trafficking (EspG and NleA; Shenoy, Furniss, Goddard, & Clements, 2018), actin dynamics (Tir, TccP, Map, EspF, EspH, EspM; Shenoy et al., 2018), cell cycle (Cif; Samba-Louaka, Taieb, Nougayrède, & Oswald, 2009), inflammatory signalling pathways (NleC, NleE; Gan, Giogha, & Hartland, 2021) and cell death, including apoptosis (EspL, EspF, EspH, NleA, NleB, NleD, NleF and NleH; Gan et al., 2021; Wong Fok Lung, Pearson, Schuelein, & Hartland, 2014).

Apoptosis is a tightly regulated process. It can occur either via extrinsic (e.g., Fas ligand, TRAIL) or intrinsic (e.g., release of Ca^2+^ from the ER) pathways, which converge on activation of the executioner caspases. In HeLa cells, activation of apoptosis via the extrinsic or the intrinsic pathway leads to oligomerisation of the Bcl-2 effectors Bcl-2-associated x protein (Bax) and Bcl-2 antagonist killer 1 (Bak), which induce mitochondrial outer membrane permeabilisation (MOMP), release of pro-apoptotic proteins (e.g., cytochrome *c*, SMAC/Diablo), formation of the apoptosome, cleavage of pro-caspase-9 and activation of the executioner caspase-3 and caspase-7 (reviewed in Bock & Tait, 2020). X-linked inhibitor of apoptosis (XIAP), which is an E3 ubiquitin ligase, is considered to be the most potent member of the inhibitor of apoptosis (IAP) family (Schile, García-Fernández, & Steller, 2008). XIAP inhibits apoptosis by impeding the active site of caspase-3, caspase-7 and caspase-9 (Eckelman, Salvesen, & Scott, 2006). In addition, HCLS1-associated protein X-1 (HAX-1) protects cells from various pro-apoptotic stimuli (Jing et al., 2011; Kang et al., 2010). HAX-1 regulates Ca^2+^ released from the ER via phospholamban and Sarco/Endoplasmic Reticulum Ca^2+^-ATPase2 (SERCA2; Larsen et al., 2020; Vafiadaki et al., 2007, 2009) inhibits caspase-9 (Shaw & Kirshenbaum, 2006) and binds XIAP, reducing its ubiquitination and subsequent proteasomal degradation (Kang et al., 2010).

Several T3SS effectors expressed by A/E pathogens have been shown to induce apoptosis (EspH and EspF). However, infection with these pathogens does not lead to cell death as they also employ a number of T3SS effectors to inhibit both intrinsic and extrinsic apoptosis (Wong Fok Lung et al., 2014). NleH1 and NleH2, which are conserved in both EPEC and EHEC, provide protection against a wide range of pro-apoptotic stimuli including staurosporine (STS), tunicamycin (TUN) and brefeldin A (Hemrajani et al., 2010; Wong, Clements, Raymond, Crepin, & Frankel, 2012). NleF inhibits FasL-induced apoptosis by binding caspases-4, -8 and -9 and inhibiting their proteolytic activity (Blasche et al., 2013; Pollock et al., 2017). Infection of cells with an EPEC E2348/69 mutant (ICC303), missing *nleH1, nleH2* and not expressing *nleF*, is highly cytotoxic resulting in rapid apoptosis leading to cell detachment which can be complemented by plasmids encoding either NleH1 or NleH2 or by the pan caspase inhibitor z-VAD (Hemrajani et al., 2010). The extrinsic apoptotic pathway (FAS ligand-induced) is also blocked by NleB1, an N-acetylglucosaminetransferase that specifically modifies Arg 117 in the death domain of FADD (Pearson et al., 2013; S. Li et al., 2013). EspL blocks caspase-independent necroptosis by cleaving RHIM (receptor-interacting protein homotypic interaction motif) containing adapter proteins (Pearson et al., 2017) and NleD, a Zn-dependent endopeptidase, inhibits apoptosis by specifically cleaving the activation loop of JNK (Baruch et al., 2011). Therefore, prolonging host cell survival appears to be an important virulence strategy of A/E pathogens.

The gene encoding the effector EspO is duplicated in the genome of EHEC O157:H7 strain EDL933, annotated as *espO1* and *espO2*. In C. *rodentium, espO* is present as one copy, while *espO* is a pseudogene in the prototype EPEC strain E2348/69 (Iguchi et al., 2009). Kim and colleagues reported that OspE of *Shigella* spp., Osp1_STYM_ of *Salmonella enterica* serovar Typhimurium and EspO belong to the same effector family and interact with ILK (Kim et al., 2009). A tryptophan residue at position 68 (W68) of OspE, or at position 77 of EspO (W77) is essential for the interaction of OspE and EspO with ILK (Kim et al., 2009). OspE localizes to focal adhesions (FAs), where it interacts with ILK, inhibits cell detachment and promotes *Shigella* attachment and cell survival (Kim et al., 2009; Miura et al., 2006). EHEC EspO2, which also binds ILK, can also block FAs disassembly by inhibiting the guanine nucleotide exchange factor (GEF) activity of the EHEC effector EspM2 (Morita-Ishihara et al., 2013). More recently, infection of mice with a *C*. *rodentium* mutant lacking EspO resulted in reduced proliferation of intestinal epithelial cells (IEC) and altered IEC signalling, leading to reduced neutrophil recruitment to the bacterial attachment site (Berger et al., 2018). At late stages of infection, a *C*. *rodentium* mutant lacking EspO also resulted in higher bacterial burden (Ale et al., 2017). In this paper, we report a new role for the EspO effector family, which is to inhibit intrinsic apoptosis via its interaction with the anti-apoptotic protein HAX-1.

## 2 Results

### 2.1 HAX-1 is an interaction partner of EspO1, EspO2, OspE and Osp1_STYM_

**Figure 1:**
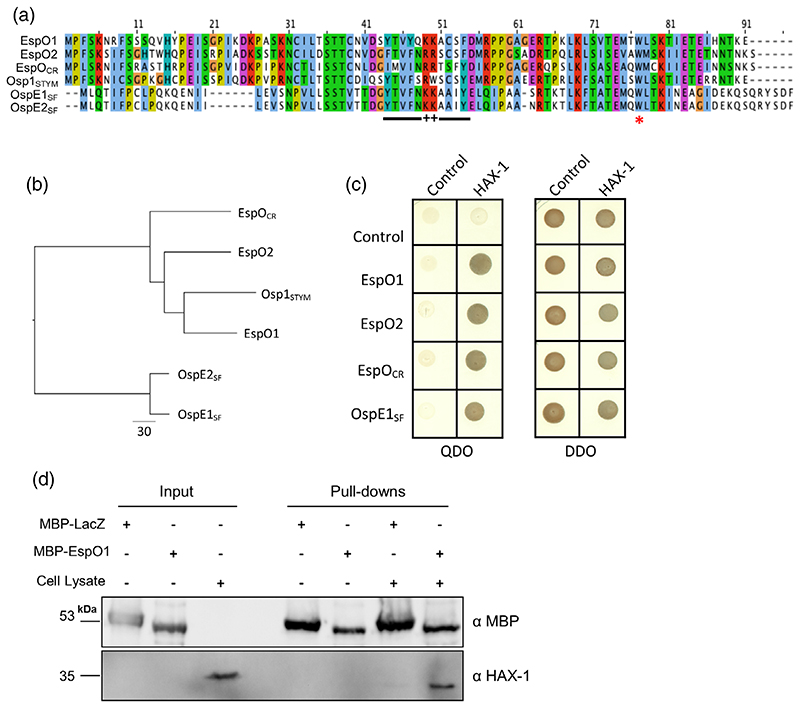
EspO and OspE bind HAX-1. (a) Sequence alignment of the EspO family of effectors. EspO1 and EspO2 from EHEC EDL933, EspO_CR_ from *Citrobacter rodentium* ICC180, OspE1_SF_ and OspE2_SF_ from *S. flexneri* strain M90T and Osp1_STYM_ from S. *typhimurium* strain SL1344 were aligned using CLUSTAL Omega. Predicted β-strands at aa 44-48 and 51-54 of EspO1 are underlined, + indicates the positively charged residues at aa 49/50 and * indicates the conserved tryptophan. (b) A phylogenetic tree of the EspO family members shown in (a), constructed using ClustalOmega with neighbour joining using BLOSUM62 and prepared with FigTree. (c) Yeast AH109 co-transformed with pGBKT7-espO homologues and pGADT7-HAX-1 grew on selective medium (QDO), indicating a protein interaction whereas yeast co-transformed with pGBKT7-espO homologues and empty pGADT7 did not. Growth on non-selective media (DDO) indicated a successful plasmid co-transformation. (d) HEK293T whole cell extracts were incubated with purified and immobilised MBP-EspO1, or MBP-LacZ as a negative control. HAX-1 is specifically pulled down with MBP-EspO1 but not MBP-LacZ

EspO belongs to a family of effectors consisting also of *Shigella* OspE (which is duplicated as OspE1_SF_ and OspE2_SF_ in *Shigella flexneri* and some *Shigella boydii* strains or present as a single intact gene in *Shigella sonnei* and *dysenteriae*) and *S*. Typhimurium Osp1_STYM_ (Figure 1a; Kim et al., 2009). Osp1_STYM_ is the closest orthologue to EspO1 within this family and OspE1_SF_ and OspE2_SF_ are the most distantly related (Figure 1b).

EspO is a pseudogene in the prototype EPEC strain E2348/69. To determine if *espO* genes are present in clinical EPEC isolates, we screened 73 EPEC strains. Ten (13.6%) isolates contained the *espO1* gene (serotypes O111:H-, O154:H9, O85:H-, O123:H- (2 isolates), ONT:H7, O55:H- (2 isolates), O55:H7, O111:H9), five (6.8%) isolates contained the *espO2* gene (O26:H-, O55:H6, O55:H7 (3 isolates)) and four (5.5%) isolates contained both *espO1* and *espO2* genes (ONT:H-, O119:H9, O49:H-, O26:H11).

In order to identify whether there are additional EspO interaction partners than the previously described ILK, we performed a yeast two-hybrid (Y2H) screen, using EspO1 as bait and human cDNA library as prey. This screen identified HAX-1 as a putative EspO1 interacting protein (19 of 27 yeast clones). We further confirmed the interaction of HAX-1 with EspO1 by direct Y2H and demonstrated that EspO2, EspO_CR_, OspE1_SF_ (Figure 1c) and Osp1_STYM_ (Figure S1) also interacted with HAX-1. The interaction between EspO1 and HAX-1 was validated by pull down assays using MBP-EspO1 and HEK293 cell lysates (Figure 1d). While a very faint band corresponding to HAX-1 can be observed in the pull down using MBP-lacZ, this band is much stronger in the pull down using MBP-EspO1 confirming an interaction between EspO1 and HAX-1. As the Y2H showed that all family members interacted with HAX-1, we mainly focused our characterisation on EspO1.

**Figure 2:**
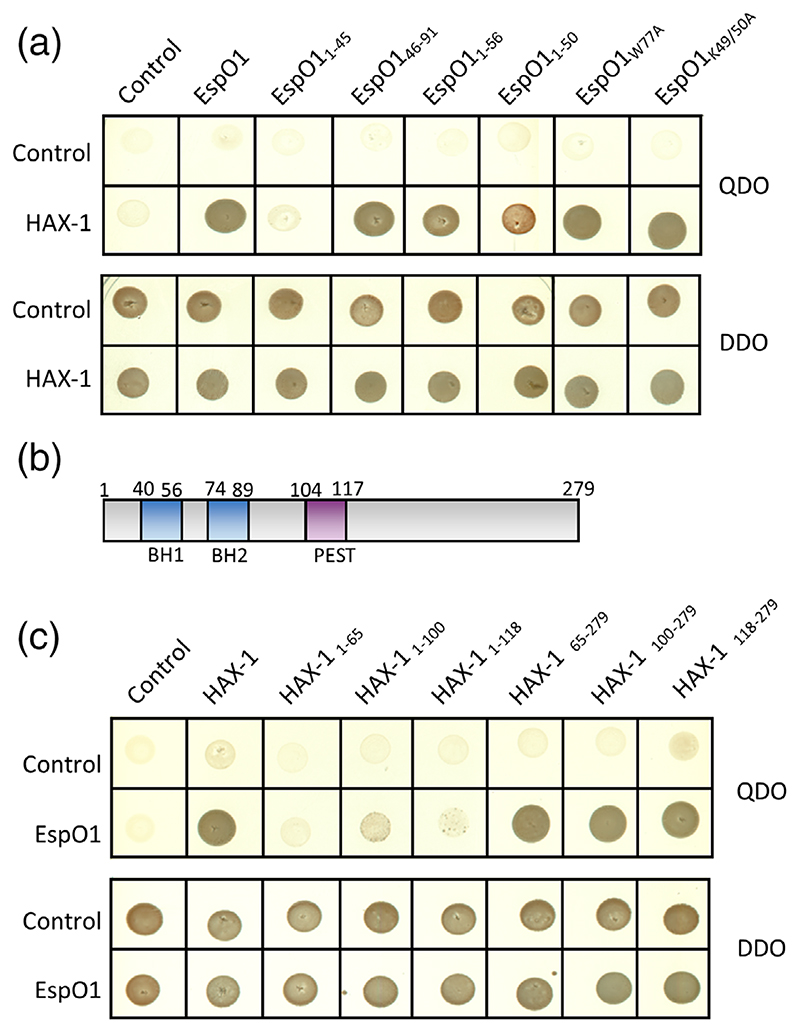
Mapping the EspO1-HAX-1 interaction sites. (a) Yeast co-transformed with HAX-1 and the EspO truncations 46-91, 1-56 and 1-50aa grew on selective media (QDO); yeast co-expressing HAX-1 and the EspO truncation 1-45aa or containing the EV (control) did not grow on selective media. Growth on non-selective media (DDO) indicated a successful plasmid co-transformation for all pairs. (b) Schematic representation of HAX-1 with Bcl-2 homology (BH) 1 and 2, and PEST domains indicated. (c) AH109 co-expressing EspO1 and the C-terminal part of HAX-1 (65-279, 100-279 or 118-279aa) grew on selective media (QDO) while yeast expressing the N-terminal region of HAX-1 (1-65, 1-100 or 1-118) did not. Growth on non-selective media (DDO) indicated a successful plasmid cotransformation for all pairs

3D structure prediction using Phyre2 (Kelley, Mezulis, Yates, Wass, & Sternberg, 2015) revealed that EspO1 does not resemble any known structures in the database. However, the internal (26-62aa) region of EspO1 was predicted with low confidence to adopt an immunoglobulin-like beta-sandwich fold which suggests that EspO1 contains 2 short β-sheets (44-48aa and 51-54aa) connected by 2 positively charged residues (K or R) in the center of the protein (indicated in Figure 1a), flanked by regions of unknown structure at the N and C termini. In order to identify the HAX-1-binding site within EspO1, we generated constructs expressing EspO1 residues 1-45, 1-50, 1-56 and 46-91, which were used as baits in direct Y2H binding assays. The smallest fragment of EspO that allowed growth on selective media indicating full binding capacity spanned residues 46-91 (Figure 2a). Consistent with this observation residues 1-45 were unable to support growth indicating these are not involved in the interaction with HAX-1. The two positively charged residues (aa 49 and 50) are not involved in the interaction as EspO1_K49/50A_ still interacts with HAX-1 (Figure 2a). Most importantly, the conserved tryptophan required for interaction with ILK was not required for the interaction with HAX-1 as EspO1_W77A_ also interacted with HAX-1 by direct Y2H (Figure 2a).

We next mapped the EspO1-binding site within HAX-1 using direct Y2H by generating a series of HAX-1 truncations. HAX-1 is a largely disordered protein that adopts partial folding in lipid membranes (Larsen et al., 2020). HAX-1 contains 2 Bcl-2 homology (BH) domains, BH1 (41-56aa) and BH2 (74-89aa), a PEST sequence (104-117aa) required for proteasomal degradation and a C-terminal region (118-279aa), which mediates interactions with multiple partners (Figure 2b; Fadeel & Grzybowska, 2009; B. Li et al., 2012). This direct Y2H revealed that, like many HAX-1 interacting partners, EspO binds within the C-terminal (118-279 aa) region of HAX-1 (Figure 2c).

### 2.2 EspO1 co-localizes with focal adhesions and HAX-1

**Figure 3:**
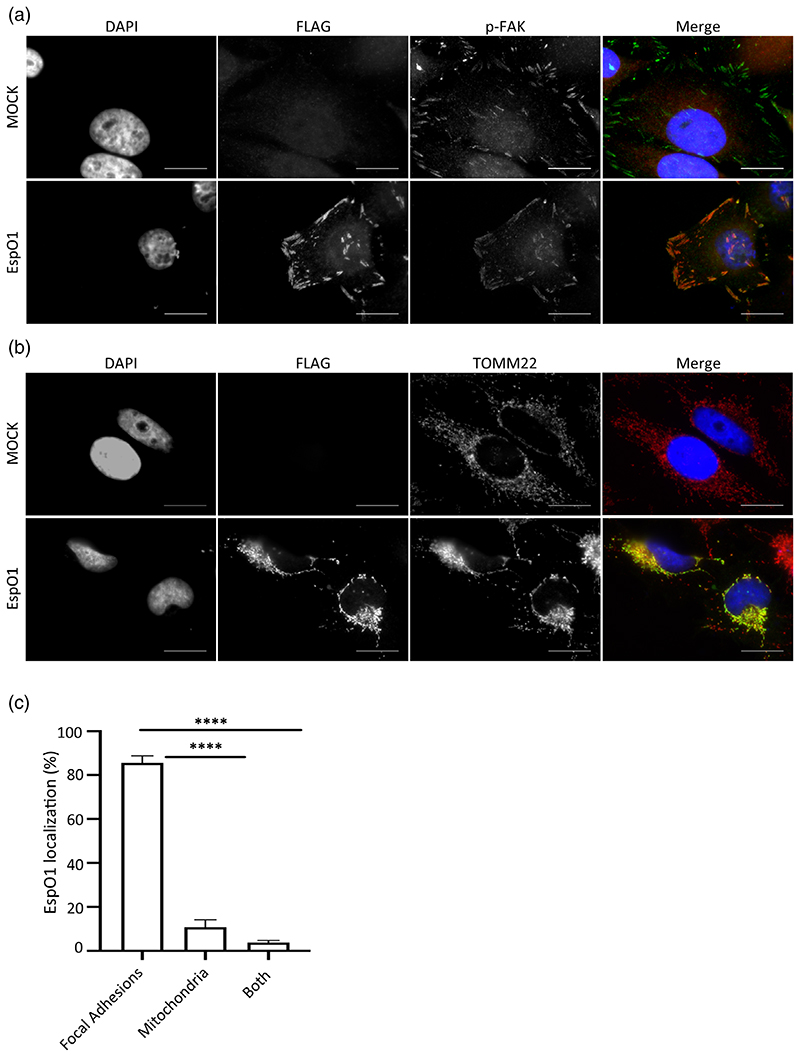
EspO1 localises to FA and mitochondria. (a) and (b) HeLa cells expressing EspO1(FLAG) were immunostained with anti-FLAG (red in merged image) to examine EspO1 localization. Focal adhesions were visualised with anti-p-FAK antibodies (a) and mitochondria with TOMM22 antibodies (b) (green in merged image). DNA was detected by DAPI (blue in merged image). Scale bar = 20 μm. (c) The percentage of cells where EspO localised to FA or mitochondria was calculated. Results are the average of 3 independent biological repeats, counting 100 cells per condition (****p < .0001)

**Figure 4:**
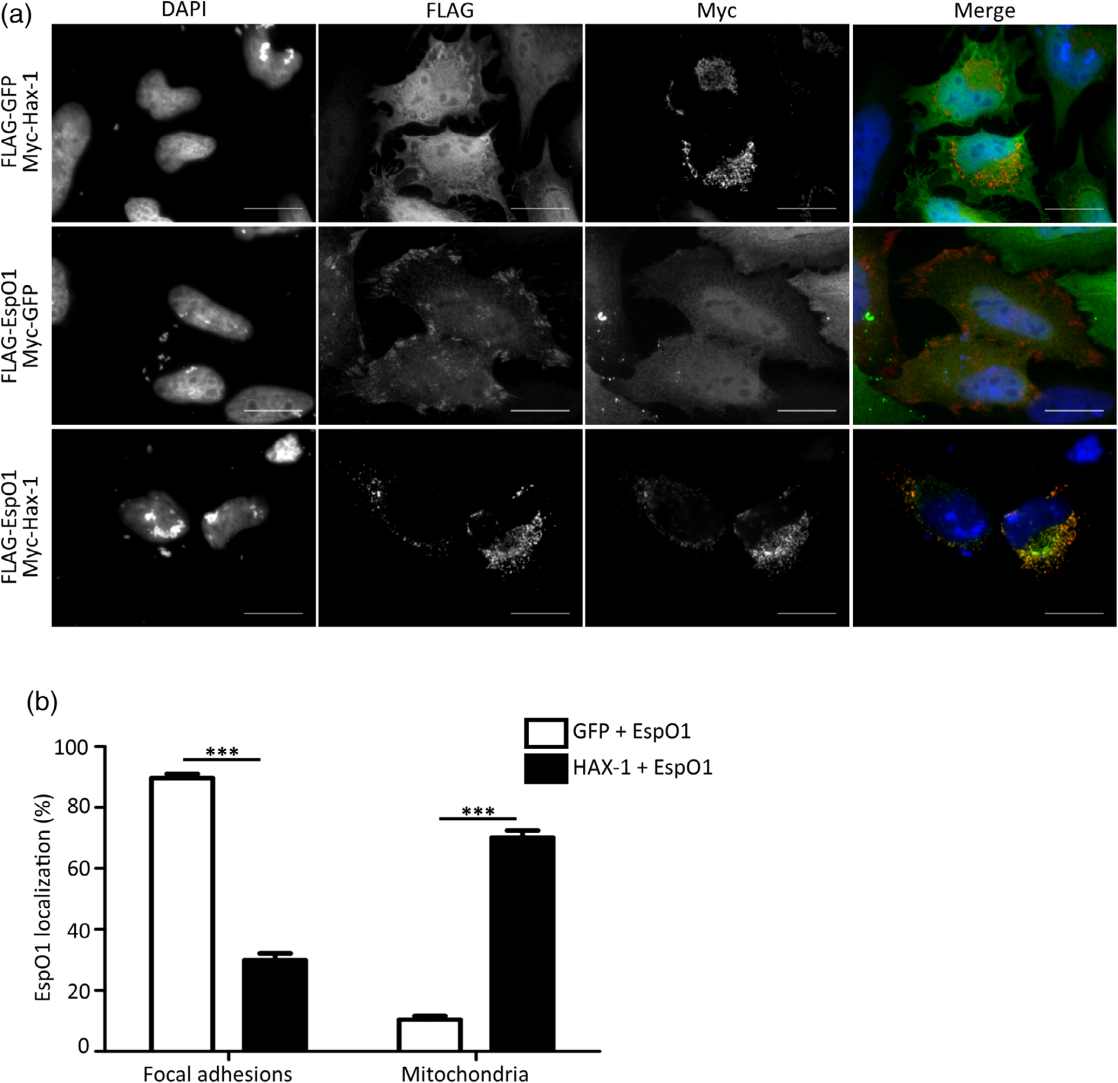
Co-localization of EspO1 with HAX-1. (a) HeLa cells expressing GFP(FLAG) and HAX-1(myc) (top row), EspO1(FLAG) and GFP(myc) (middle row), or EspO1(FLAG) and HAX-1(myc) (bottom row), were immunostained with anti-Flag and anti-Myc antibodies. Anti-FLAG staining is green, anti-myc staining is red and DNA (DAPI) is blue in the merged image. Scale bar = 20 μm. (b) The percentage of cells where EspO1 was distributed to FAs or mitochondria was calculated in cells co-expressing EspO1 with either GFP or HAX-1. When co-expressed with GFP, EspO1 was mainly distributed to FAs; however, when HAX-1 is overexpressed, EspO1 was mainly distributed to mitochondria in the majority of cells (***p < .001). Results are the average of three independent biological repeats, counting 100 cells per condition

We used immunofluorescence microscopy (IF) to determine the localisation of ectopically expressed EspO1 in HeLa cells. This revealed that EspO1 localized mainly to FAs (85.5% of cells) or the mitochondria (10.7% of cells), as it co-localized with either phosphorylated Y397 focal adhesion kinase (p-FAK) or the mitochondrial membrane protein TOMM-22 (Figure 3). In a small percentage of cells EspO could be seen in both FAs and mitochondria (3.8%). To study the co-localisation of EspO1 with HAX-1, we ectopically expressed EspO1(FLAG) and HAX-1 (myc). GFP(myc) and GFP(FLAG) were used as controls. IF analysis revealed co-localisation of EspO1 and HAX-1 (Figure 4a). Interestingly, co-expression of HAX-1 with EspO1 appeared to shift the distribution of EspO1 from predominantly FA-localized to predominantly mitochondria-localized. To verify the dependence of EspO1 localisation on HAX-1 expression, the localisation of ectopically expressed EspO1 (FLAG) was quantified in the presence or absence of ectopically expressed HAX-1(myc) (Figure 4b). When co-expressed with GFP, EspO1 staining closely resembled that of FAs (89.7%), however, when co-expressed with HAX-1, EspO1 staining was observed in structures that resembled mitochondria (70.2%), suggesting that increased expression of one interacting partner may significantly alter EspO1 distribution (Figure 4b).

### 2.3 EspO1 has anti-apoptotic activity

**Figure 5:**
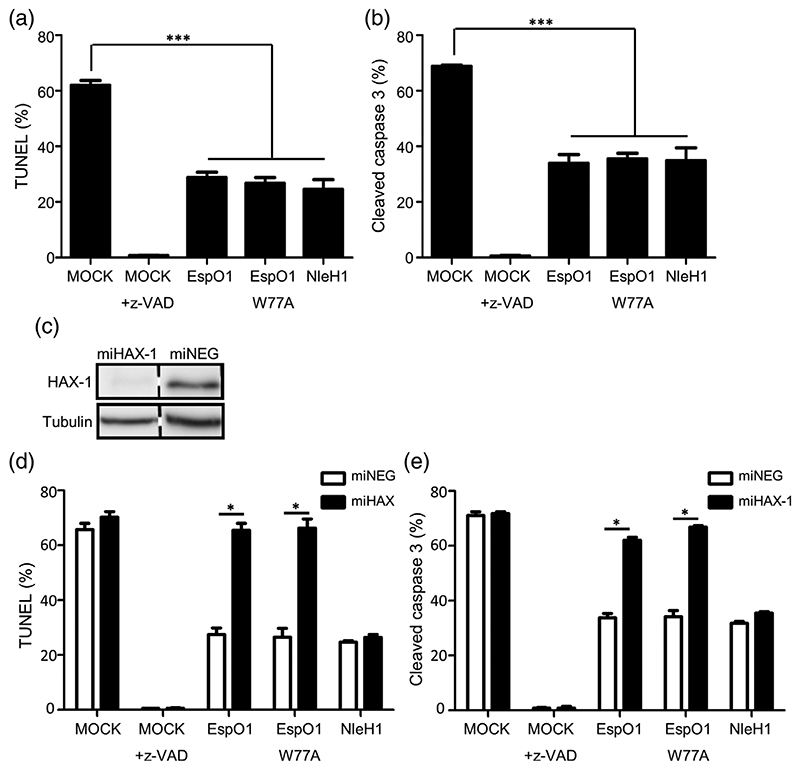
EspO inhibits STS-induced apoptosis. Cells expressing EspO(FLAG), EspO1_W77A_(FLAG) or NleH1(HA) were treated with STS and apoptosis measured by detecting DNA fragmentation (fluoroscein-12-dUTP) (a) or cleaved caspase-3 (b). Ectopic expression of EspO1, EspO1_W77A_, NleH1 or z-VAD treatment prevented DNA fragmentation and cleavage of procaspase-3 induced by STS compared with mock-transfected cells. Results are the average of three independent biological repeats, counting 100 cells per condition. (***p < .001). Representative images can be seen in Figure S2a,b. (c) Cell lysates from miHAX-1 and the parental miNEG cells were analyzed by Western blot with HAX-1 and tubulin-control antibodies to demonstrate HAX-1 knockdown. (d) and (e) miNEG or miHAX-1 cells expressing EspO(FLAG), EspO1_W77A_(FLAG) or NleH1(HA) were treated with STS and apoptosis measured by detecting DNA fragmentation (fluoroscein-12-dUTP) (d) or cleaved caspase-3 (e). The pan-caspase inhibitor z-VAD was used as apoptotic inhibitor control. The results were obtained from three independent biological repeats, counting 100 cells per condition (*p < .05)

As HAX-1 plays a role in inhibiting apoptosis (Fadeel & Grzybowska, 2009), we tested if EspO1 modulates cell death. To exclude any effect of ILK interaction EspO1_W77A_ (which interacts with HAX-1 but not ILK) was included. Cells ectopically expressing EspO1 (FLAG) or EspO1_W77A_(FLAG) were treated with STS and the level of apoptosis quantified by DNA fragmentation (TUNEL assay; Figure 5a and Figure S2a), or cleaved caspase-3 staining (Figure 5b and Figure S2b). Cells expressing NleH1(HA) or treated with z-VAD were used as controls. In both assays, EspO1 and EspO1_W77A_ significantly blocked apoptosis when compared with mock-transfected cells. This was comparable to the anti-apoptotic effect observed with NleH1 (Figure 5a,b). Similar results were obtained when apoptosis was induced by tunicamycin (Figure S2d). Importantly, cells expressing OspE1, OspE1_W68A_ and Osp1_STYM_ also inhibited STS and tunicamycin-induced apoptosis to a similar level as cells EspO1, EspO1_W77A_ or NleH1 indicating that the anti-apoptotic function of EspO is conserved (Figure S2c,d). In contrast, the inhibition of cell death was not observed in control cells expressing GFP (Figure S2c,d). These results suggest that EspO1, OspE1 and Osp1_STYM_ are anti-apoptotic effectors and that their anti-apoptotic activity is independent of the tryptophan residue implicated in ILK binding.

In order to investigate whether the anti-apoptotic activity of EspO1 was HAX-1 dependent, we utilized a HAX-1 knocked down HeLa cell line (miHAX-1; Grzybowska et al., 2013). A control cell line, transfected with the same plasmid but without a silencing sequence (miNEG), was used for comparison. Following confirmation that HAX-1 was successfully knocked down by Western blotting (Figure 5c), we ectopically expressed EspO1(FLAG), EspO1W77A (FLAG) and NleH1(HA) in miHAX-1 and miNEG cells; z-VAD was used as an additional control. As previously shown expression of EspO1, EspO1_W77A_ and NleH1 decreased STS-induced apoptosis in the control miNEG cells compared to MOCK-transfected cells when measured by TUNEL (Figure 5d) or cleaved caspase-3 staining (Figure 5e). In contrast, EspO1 and EspO1_W77A_ lost their ability to protect cells against STS treatment in the HAX-1 knocked down cells by TUNEL and cleaved caspase 3 staining (Figure 5d,e). Importantly, both assays showed that z-VAD and NleH1 were able to equally protect miHAX-1 and miNEG cells from STS-induced apoptosis (Figure 5d,e). These results show that HAX-1 is specifically involved in the anti-apoptotic activity of EspO1 but not NleH1.

### 2.4 EspO1 interacts with HAX-1 during infection

**Figure 6:**
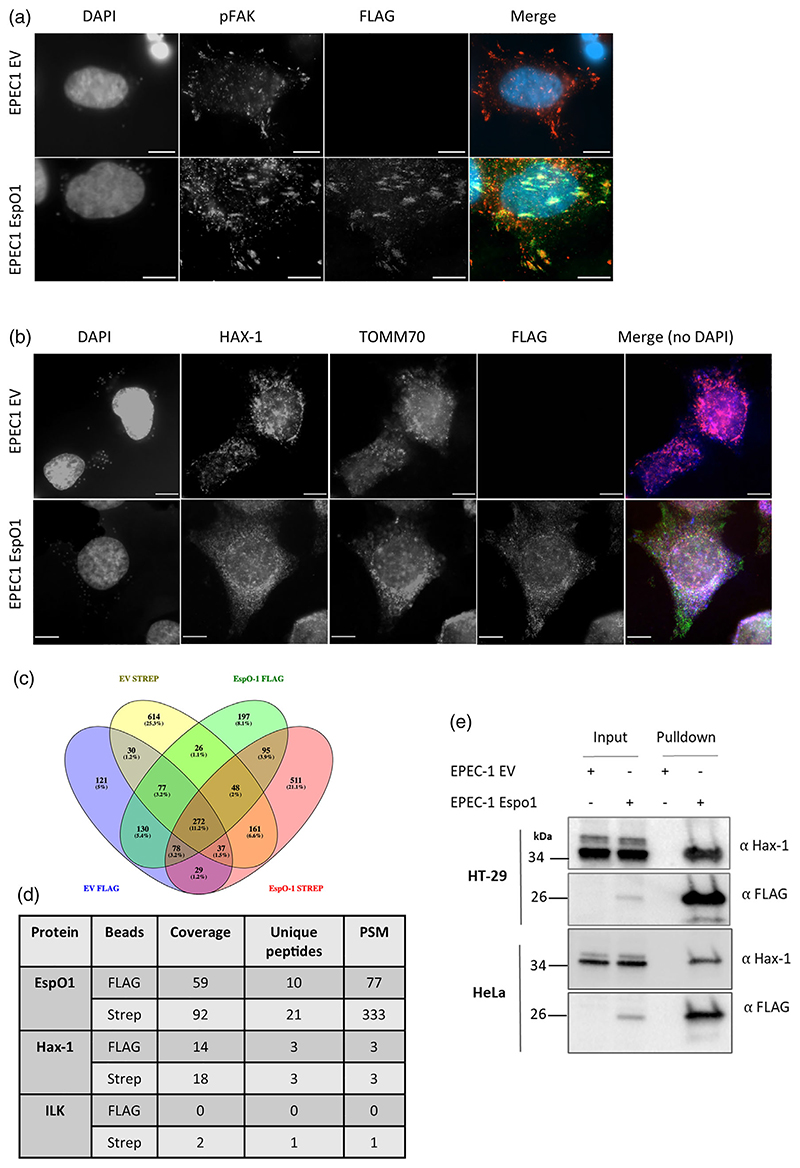
EspO1 interacts with HAX-1 during in *vitro* infection. (a) and (b) HeLa cells were infected with EPEC1 encoding either an empty vector (EV) or EspO1 (FLAG-Strep). Cells were immunostained with anti-FLAG to detect EspO1, together with anti-pFAK to detect focal adhesions (a) or anti-HAX-1 and TOMM70 to detect HAX-1 and mitochondria (b). HeLa and bacterial DNA was detected by DAPI. The merged image in (b) does not include DAPI staining for simplicity. Scale bar = 10 μm. (c) Venn diagram depicting overlap between proteins identified by MS/MS in FLAG and Strep pulldowns from HT-29 cells infected with EPEC-1 encoding either an empty vector (EV) or EspO1 (FLAG-Strep). (c) Coverage, number of peptides and peptide spectrum matches (PSMs) obtained by MS/MS for EspO1 and HAX-1 in FLAG and Strep pulldowns from HT-29 cells infected with EPEC-1 encoding EspO1 (FLAG-Strep). (d) Validation of EspO1 and HAX-1 interaction during infection by immunoblotting of FLAG-pulldown from HT-29 and HeLa cells infected with EPEC-1 encoding either an empty vector (EV) or EspO1 (FLAG-Strep)

We next aimed to investigate whether the interacting partners of EspO1 could be confirmed during infection. Identification of interacting partners of T3SS effectors can be difficult to perform during infection due to the large number of translocated effectors. To overcome this, we used EPEC1, an E2348/69 strain that has all known T3SS effectors deleted, except Tir which ensures intimate attachment of the bacterium to cells (Cepeda-Molero et al., 2017). EspO1^FLAG-STREP^ was ectopically expressed in EPEC1 and trans-located into HeLa cells during a 3 hr infection as confirmed by immunofluorescence analysis (Figure 6a,b). EspO1 can mainly be observed localizing to FA (Figure 6a) with a Pearson's correlation coefficient (*r*) of .64 between EspO-FLAG and pFAK. Occasionally, mitochondrial localisation was also observed (Pearson's *r* value of .65 between EspO-FLAG and TOMM70), along with HAX-1 colocalisation (Pearson's *r* value of .72 between EspO-FLAG and HAX-1; Figure 6b).

EspO1, delivered by EPEC1 into the colorectal cancer cell line HT-29, was then affinity purified by FLAG or Strep beads and the EspO1 interactome was identified by MS/MS after on-bead digestion. Side-by-side FLAG and Strep pulldowns were performed using EPEC1 encoding an empty vector (EV). Proteins identified in these samples were designated non-specific and removed from further analysis. Ninety-five proteins were specifically identified in both FLAG and Strep pulldowns with EspO1 (Figure 6b and Table S4). The MS/MS parameters for all proteins identified in both controls and pulldowns have been listed in Tables S5–S8. As expected, the bait EspO-1 was**FIGURE 3** Legend on next page.one of the most highly enriched proteins identified with 77 and 333 peptide spectrum matches (PSMs) in the FLAG and Strep pulldowns, respectively (Figure 6c). HAX-1 was also identified in both pull-downs, while ILK was identified as an interacting partner in the Strep but not the FLAG pull-down. The interaction of EspO1 and HAX-1 was further confirmed in a targeted FLAG pull-down by immunoblotting for HAX-1 following infection in HT-29 and HeLa cells (Figure 6d).

### 2.5 EspO1 inhibits cell death during infection in a HAX-1-dependent manner

**Figure 7:**
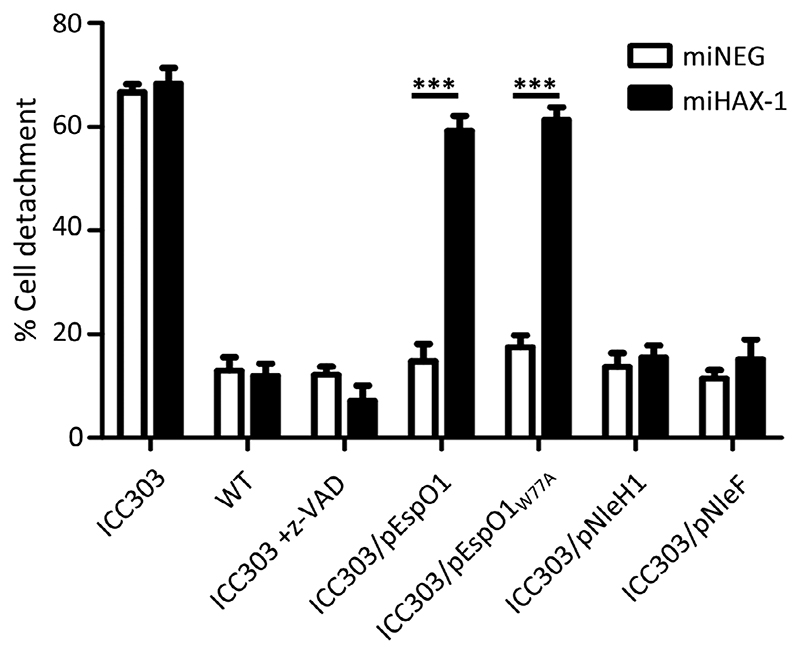
EspO prevents cell detachment induced by EPEC ICC303. Quantification of cell detachment following infection of miHAX-1 and miNEG HeLa cells with WT EPEC, EPEC *ΔnleH1ΔnleH2ΔnleF* mutant (ICC303), and ICC303 complemented with EspO1, EspO1_W77A_, NleH1 or NleF or treated with z-VAD. All complemented strains can reduce cell detachment in miNEG cells but EspO1 and EspO1_W77A_ lose their protective capability in miHAX-1 cells. The results were obtained from 3 independent biological repeats (*p < .05)

Finally, to investigate whether EspO1 was also protecting against cell death during infection, we utilized a cell detachment assay. EHEC encodes two EspO paralogs along with the anti-apoptotic effectors NleH1, NleH2 and NleF. Therefore, it is not surprising that EHEC mutants missing either *espO1/espO2, nleH1/nleH2* or *nleF* do not induce cell detachment (Figure S3). In order to circumvent the problem of creating an EHEC *espO1, espO2, nleH1, nleH2, nleF* mutant, we used EPEC strain ICC303 (a double *nleH1* and *nleH2* E2348/69 deletion mutant, which has a polar effect on expression of *nleF)*. ICC303 is highly cytotoxic, causing cell death leading to cell detachment (Hemrajani et al., 2010). The cell detachment phenotype of this strain could be complemented by expression of NleH1 from a plasmid or by z-VAD (Hemrajani et al., 2010). Accordingly, we investigated if EspO1 could also prevent cell detachment following infection of HeLa cells with ICC303 and if so, whether this was dependent on HAX-1. HeLa miNEG and miHAX-1 cells were infected for 90 min with ICC303, or ICC303 expressing EspO1, EspO1_W77A_ or NleH1 or NleF as controls, before quantification of the remaining attached cells. Treating ICC303-infected cells with z-VAD was used as an additional control. Consistent with previous results, infection of miNEG with ICC303 resulted in significant cell detachment (67%) compared with cells infected with wild type (WT) EPEC (13%; Figure 7). The introduction of EspO1, EspO1_W77A_, NleH1 or NleF or z-VAD treatment of ICC303 significantly reduced cell detachment to 15%, 18%, 14% 12% and 16%, respectively (Figure 7). However, in the absence of HAX-1 (miHAX-1 cells), ICC303 expressing EspO1 and EspO1_W77A_ were no longer able to prevent cell detachment (59% and 61% cell detachment, respectively), while NleH1, NleF and z-VAD treatment all retained their protective capabilities (15% cell detachment; Figure 7). This suggests that EspO1 inhibits cell detachment during infection in a HAX-1-dependent/ILK independent manner, while NleH1 and NleF operate independently of HAX-1.

## 3 Discussion

Although the prototype EPEC strain E2348/69 contains a pseudo *espO* gene, 25% of the clinical EPEC isolates tested contained *espO1*, *espO2* or both. EHEC O157:H7 strains encode both *espO1* and *espO2*. This suggests that the EspO effector family is an important T3SS virulence factor amongst pathogenic *E*. *coli* as well as *Shigella* and *S*. *typhimurium*.

EPEC and EHEC use T3SS effectors to control host cell functions, including cell-cycle, -death and -survival. Although several effectors (e.g., EspH, EspF) trigger cell death (Crane, McNamara, & Donnenberg, 2001; Nougayrède & Donnenberg, 2004; Wong et al., 2012), infected cells do not undergo apoptosis (Wong Fok Lung et al., 2014). This is due in part to the alleviation of cytotoxic effects by NleH1 and NleH2 (which bind Bax inhibitor 1; Hemrajani et al., 2010), NleF, which binds and inhibits caspases-9, -8 and -4 (Blasche et al., 2013), NleB which inhibits death receptor signalling (Li et al., 2013; Pearson et al., 2013), EspL that inhibits caspase-independent cell death (Pearson et al., 2017) as well as the T3SS gate-keeper EspZ (Berger et al., 2012; Shames et al., 2010). In this paper, we have shown that the EspO effector family are additional anti-apoptotic effectors that inhibit cell death through an interaction with the anti-apoptotic protein HAX-1.

Using non-targeted pull downs during infection, we confirmed that HAX-1 was part of the EspO1 interactome when EspO is delivered by the T3SS during infection. In addition to HAX-1, 2 other proteins involved in apoptotic signalling were found in the EspO interactome in the STREP pulldown; p53 and BH3 interacting domain death agonist (BID). While these proteins are unlikely to directly interact with EspO they suggest that through an interaction with HAX-1, EspO can modulate apoptotic signalling complexes to alter the cellular response to apoptotic stimuli. Interestingly ILK that was previously reported to interact with the EspO family of effectors (Kim et al., 2009) was identified only in one of the EspO1 pull-downs. This may reflect differing levels of protein abundance between HAX-1 and ILK or a less stable interaction between ILK and EspO1. HAX-1 is a part of the ILK interactome (Dobreva, Fielding, Foster, & Dedhar, 2008), which could suggest that EspO1 and its paralogs target the HAX-1-ILK complex through multiple interactions.

OspE1 interacts with ILK at FAs where it stabilizes cell-matrix adhesion sites and inhibits cell detachment during *Shigella* infection (Kim et al., 2009). We demonstrated that the ability of EspO1 to prevent cell detachment during EPEC infection occurred independently of the tryptophan residue implicated in the interaction with ILK. The ability of OspE1 to promote cell survival was believed to be mediated via ILK through its roles in cell adhesion, cell spreading, stress fiber formation and numerous signalling pathways such as Akt and Wnt (Kim et al., 2009; Petit & Thiery, 2000; Wu & Dedhar, 2001). Here, we report that OspE binds HAX-1 and blocks STS-induced apoptosis independently of the interaction with ILK. In addition to OspE *Shigella* uses other effectors to promote cell survival including IpgD (Puhar, Tronchère, Payrastre, Tran Van Nhieu, & Sansonetti, 2013), OspD2 (Mou, Souter, Du, Reeves, & Lesser, 2018) and OspC3 (Kobayashi et al., 2013). Like *Shigella*, *Salmonella* also uses multiple effectors to subvert cell survival signalling including SseK1 and K3 (Günster, Matthews, Holden, & Thurston, 2017), SopB (Knodler, Finlay, & Steele-Mortimer, 2005) and AvrA (Zhang et al., 2015). We demonstrate that the S. *typhimurium* effector Osp1_STYM_, which interacts with ILK (Kim et al., 2009), also binds HAX-1 and inhibits STS-induced apoptosis.

HAX-1, which is a ubiquitously expressed anti-apoptotic protein, interacts with numerous cellular partners including XIAP, caspase-3, caspase-9, the mitochondrial serine protease Omi/HtrA and SERCA2 in the endoplasmic reticulum (ER; Cilenti et al., 2004; Kang et al., 2010; Lee et al., 2008; Shaw & Kirshenbaum, 2006; Vafiadaki et al., 2009). HAX-1 has 8 different splicing variants (Trebinska et al., 2010); only variant I has been extensively studied and is reported to localize at mitochondria (Koontz & Kontrogianni-Konstantopoulos, 2014). In this study, we found that ectopically expressed EspO1 localizes to both FAs and to the mitochondria, but overexpression of HAX-1 shifted the localisation of EspO1 to the mitochondria. SERCA2, which actively pumps Ca^2+^ into the ER from the cytosol, is regulated by an interaction with HAX-1 (Vafiadaki et al., 2009). Overexpression of HAX-1 leads to SERCA2 proteasomal degradation, resulting in diminished ER Ca^2+^ content and protection of mitochondria from Ca^2+^ overload (Vafiadaki et al., 2009). HAX-1 is degraded by Omi/HtrA2 in an early stage of apoptosis (Cilenti et al., 2004). At later stages, SMAC/Diablo is released from the mitochondria and binds XIAP, leading to the liberation of active caspases from XIAP and to XIAP degradation (Srinivasula et al., 2001). The inhibition of Omi/HtrA2, as well as over expression of HAX-1 or XIAP, protect cells from apoptotic stimuli (Hegde et al., 2002; Kang et al., 2010; Trebinska, Högstrand, Grandien, Grzybowska, & Fadeel, 2014). In this paper, we have shown that expression of EspO1 has a similar effect.

We have established that EspO1 exerts its anti-apoptotic effect both during ectopic expression and bacterial infection, in a HAX-1-dependent manner. While EspO and its family members bind both ILK and HAX-1, EspO1_W77A_, which cannot bind ILK, is able to protect cells against cell death. Importantly, the anti-apoptotic activities of NleH1 and NleF are HAX-1 independent. Although the exact mechanism through which the EspO family prevents cell death is not known, it is possible that they facilitate the stability of HAX-1 and/or XIAP or modulates the levels of Ca^2+^ in the ER to protect cells against intrinsic apoptosis. The fact that the extracellular pathogens EHEC and CR and the invasive pathogens *Shigella* and S. *typhimurium* use T3SS effectors to target HAX-1 signalling suggests that it plays a central role in pathogen host interactions.

## 4 Experimental Procedure

### 4.1 Strains, growth conditions and reagents

*E*. *coli* strains used in this study are listed in Table S1. ICC303, constructed as a double *nleH1*/*nleH2* mutant, was later found to have a polar effect on expression of NleF and therefore is functionally a triple *nleH1/nleH2/nleF* mutant. E. *coli* was cultured in Luria Broth at 37°C, 200 rpm with appropriate antibiotics, ampicillin 100 μg/ml, kanamycin 50 μg/ml and chloramphenicol 25 μg/ml. *Saccharomyces cerevisiae* was grown either at 30°C, 200 rpm in YPD media or 30°C on Sabouraud agar (QDO) with amino acids dropout if required (DDO); Leu, Trp, Ade, His. Reagents were purchased from Sigma-Aldrich, unless stated otherwise.

### 4.2 Plasmid construction

Plasmids and primers used in this study were listed in Tables S2 and S3, respectively. *espO1* full length were amplified from *E*. *coli* O157: H7 strain EDL933 genomic DNA by PCR using KOD Hot Start polymerase (Novagen) and cloned in pGBKT7 using primer FE1/RE1 generating pICC1919, pSA10 using primer FE7/RE7 generating pICC1378, pcDNA-NTAP using primer FE6/RE6 generating pICC1927, pMAL-c2X using primer FE8/RE8 generating pICC2025 or pRK5-myc using primer FE9/RE9 generating pICC1931. *espO1* truncations 1-45aa, 46-91aa, 1-50aa and 1-56aa were amplified from EDL933 genomic DNA using primers FE1-RE2, FE2/RE1, FE1/RE3 and FE1/RE4, respectively, and cloned in pGBT7 generating pICC2020 pICC1929, pICC2021 and pICC1930, respectively. *espO2* were amplified from EDL933 genomic DNA and cloned in pGBKT7 using primer F2/R2 generating pICC2019 or pRK5-myc using primer F3/R3 generating pICC2026. *espO* were amplified from CR ICC180 genomic DNA and cloned in pGBKT7 using primer FC1/RC1 generating pICC1920 or pRK5-myc using primer FC2/RC2 generating pICC2027. *ospE1* were amplified from *Shigella flexneri* M90T genomic DNA and cloned in pGBKT7 using primer FO1/RO1 generating pICC1921 or pRK5-myc using primer FO2/RO2 generating pICC2029. *ospE and nleH1* were amplified from S. *typhimurium* SL1344 and EPEC E2348/69 genomic DNA, respectively, and cloned in pRK5-myc using primer FT1/RT1 and FN1/RN1 generating pICC2028 and pICC2018, respectively. Tryptophan mutant W68A, W77A, K49/50A were generated with QuickChangeII kit (Stratagene) using primer 68AF/68AR, 77AF/77AR and KK1/KK2, respectively. HAX-1 full length and truncations 1-65aa, 1-100aa, 1-118aa, 65-279aa, 100-279aa and 118-279aa were amplified from pCMV-HAX-1 (Vafiadaki et al., 2009) using primers HF1/HR1, HF1/HR2, HF1/HR3, HF1/HR4, HF2/HR1, HF3/HR1, HF3/HR1 and HF4/HR1, respectively, and cloned in pGBT7 generating pICC1932, pICC1925, pICC1933, pICC2016, pICC1934, pICC1923 and pICC1928. All constructs were verified by DNA sequencing (GATC biotech). EPEC clinical isolates were screened for the presence of *espO1* and *espO2* by PCR using pair primers FE9/RE9 and F3/R3.

EHEC EspO1 with 3-x FLAG-TwinStrepII tag at C-terminus was synthesized by GeneArt (Thermo Fisher). This was inserted in pSA10 vector using restriction digestion and ligation. The plasmid with EspO1 or an EV was electroporated in EPEC-1 to generate EPEC-1 EspO1 (FLAG-Strep) and EPEC-1 EV.

### 4.3 Yeast transformation

S. *cerevisiae* wild-type yeast strain AH109 was grown overnight at 30°C in YPDA broth and then centrifuged at 4000 rpm for 15 min. The pellet was washed twice with sterile water and resuspended in the transformation mix containing 50% w/v PEG 3350, 36 μl 1M LiAc, 50 μl Herring sperm DNA (2 mg/ml), 34 μl sterile H_2_O and 1 μg of the appropriate plasmid to be transformed. The mixture was incubated at 42°C for 30 min, centrifuged and resuspended in sterile H_2_O and 100 μl plated onto appropriate selective plates.

### 4.4 Yeast two-hybrid screening

A yeast two-hybrid screen was performed using EHEC EDL933 EspO1 as a bait with the Matchmaker Pretransformed Normalised HeLa cDNA library (Clontech) according to the manufacturer's instructions. Positive clones were selected by plating on high-stringency media lacking Trp, Leu, His and Ade with X-alpha-gal (quadruple dropout media or QDO). Colonies were re-streaked on QDO and inserts amplified using the AD-LD primers (Clontech). The inserts were sequenced and identified using Blastn and Blastx.

### 4.5 Cell culture

HeLa cells (ATCC) were maintained in low glucose Dulbecco's Modified Eagle Medium (DMEM) supplemented with heat-inactivated fetal calf serum (10% vol/vol; FCS, Gibco), 2 mM GlutaMAX (Invitrogen) and 0.1 mM nonessential amino acids at 37°C under 5% CO_2_ atmosphere. For HeLa miNEG and miHAX-1 (Grzybowska et al., 2013), media was supplemented with 7.5 μg/ml blasticidin (InvivoGen), penicillin and streptomycin (Sigma). HEK293 cells were grown at 37°C in 5% CO_2_ shaking 120 rpm in Invitrogen Freestyle medium (contains Pluronic F-68 and HEPES) supplemented with 1% bovine serum. HT-29 was cultured in Roswell Park Memorial Institute (RPMI) medium (Sigma) with 10% (v/v) foetal bovine serum (FBS) (Gibco), 10 mM N-2-hydroxyethylpiperazine-N-2-ethane sulfonic acid (HEPES; Sigma), 1 mM sodium pyruvate (Sigma), 2 mM Glutamax (Gibco) and 2500 μg/ml glucose (Sigma).

### 4.6 Protein purification and pull-down

MBP-EspO1 and MBP-lacZ (control) were purified from *E*. *coli* strain Top10 following induction with 0.3 mM IPTG for 2 hr cells at 37°C. The pellet was re-suspended in column buffer (20 mM Tris–HCL, 200 mMNaCl and 1 mM EDTA), lysed by sonication for 5 min and centrifuged at 9000*g* for 30 min. The supernatant containing soluble protein was incubated with an amylose resin and washed three times with column buffer containing 1 mM DTT. The resin was then incubated with HEK293 cell lysate (2 x 10^7^ cells lysed in RIPA buffer [150 mM sodium chloride, 1.0% NP-40, 0.5% sodium deoxycholate, 0.1% SDS, 50 mM Tris—pH 8.0, with complete mini-protease and phosphatase inhibitors EDTA-free (Roche)]) overnight at 4°C. Unbound proteins were removed with three washes of column buffer and bound proteins eluted in 10 mM maltose on ice for 20 min. The samples were analyzed by SDS-PAGE and western blot analysis. Anti-MBP (NEB, E8032), anti-HAX-1 (BD Transduction Laboratories, 610825) and anti-alpha-tubulin (clone DM1A; Sigma, T6199) were used as primary antibodies diluted in 1% skim milk/PBST. Horseradish peroxidase (HRP)-conjugated goat anti-mouse IgG (Jackson ImmunoResearch, 115-035-008) diluted in 1% skim milk/PBST was used to detect the primary antibodies and HRP visualized by Ez-ECL solution (Geneflow) and captured on a LAS-3000 imager.

### 4.7 Transfection and STS induced apoptosis

HeLa cells were seeded at the concentration of 5.5 x 10^4^ cells/well in 24 well-plate containing 13 mm glass cover slips (VWR International). On the following day, cells were transfected using GeneJuice transfection reagent (Novagen/Merck) according to the manufacturer instructions. After 21 hr, the cells were washed three times in PBS and treated with 1 μM STS (Sigma) for 3 hr and 20 μM z-VAD-fmk (Promega) when required. The cells were washed three times by PBS and fixed by 3.7% paraformaldehyde (PFA) for 20 min.

### 4.8 Immunofluorescence staining and TUNEL assay

PFA fixed cells were quenched in 50 mM NH_4_Cl/PBS and perme-abilized with 0.1% Triton-X 100/PBS for 8 min. For the TUNEL assay, coverslips were treated following the manufacturer protocol. For all other immunofluorescence staining cover slips were blocked for 30 min in 2% bovine serum albumin (BSA)-PBS. Primary antibodies in 1% BSA-PBS were incubated for 45 min at room temperature, the cells washed three times with PBS, then incubated with secondary antibodies for 30 min, washed and coverslips mounted with ProLong Gold Antifade reagent (Invitrogen). Anti-Myc tag (clone 4A6; Millipore, 05-724), anti-FLAG tag (Sigma, F1804), anti-HA tag (clone16B12; Cambridge Bioscience, MMs-101P-1000), anti-pFAK (Abcam, ab4803 and ab81298), anti-TOMM22 (Abcam, ab57523), anti-TOMM70 (Atlas antibodies HPA048020), Cleaved Caspase-3 (Cell signaling, 9664) anti-GFP (Abcam, ab-290), anti-HAX-1 (Santa Cruz, sc-34273), anti-HAX-1 (R&D Systems, AF5458-SP) and anti-FLAG M2-FITC-conjugated (Sigma, F4049) were used as primary antibody. Secondary antibodies include DyLight 488-conjugated donkey anti-mouse IgG (H + L) (Jackson ImmunoResearch, 715-485-150), RRX conjugated donkey anti-mouse IgG (H + L) (Jackson ImmunoResearch, 715-295-150) and Alexa Fluor® 488 conjugated donkey anti-rabbit IgG (H + L) (Jackson ImmunoResearch, 711-545-152). 4′using the Coloc2 function in ImageJ. P,6-Diamidino-2-phenylindole (DAPI) was used to visualize cell nuclei. Immunofluorescence was visualized with a Zeiss Axio Imager immunofluorescence microscope using Zeiss AxioVision software and images were processed using ImageJ. Colocalisation analysis was quantitatively analysed using the Coloc2 function in ImageJ. Pearson's correlation coefficient was calculated for each image with Costes threshold regression. Pearson's *r* values >.6 were considered to demonstrate strong colocalisation.

### 4.9 In vitro infection and pulldown

HT-29 cells were seeded at a concentration of 0.3 x 10^6^ cells per well in a six-well plate one day before infection. EPEC-1 EV and EPEC-1 EspO1 strains were primed by diluting the overnight cultures 50x in non-supplemented Dulbecco's Modified Eagle Medium (DMEM), low glucose and incubating static at 37°C with 5% CO_2_ for 3 hr. 0.5 mM IPTG (isopropyl β-d-1-thiogalactopyranoside) was used to induce EspO1 expression from the plasmid 30 min before infection. Infection was carried out at a multiplicity-of-infection (MOI) of 50:1 for 3 hr at 37°C, 5% CO_2_. Post infection, cells were washed three times in PBS followed by incubating the cells in lysis buffer for 20 min (50 mM Tris–HCl, pH 7.4, with 150 mM NaCl, 1 mM EDTA and 1% TRITON X-100) with freshly added protease inhibitors. The lysate was collected and unlysed cells and debris removed by centrifugation at 12,000g. Anti-FLAG M2 affinity resin and MagStrep “type3” XT beads (iba) were used for pulldowns as per manufacturer's instructions. Binding to beads was performed for 4 hr at 4°C. The beads were directly processed for trypsin digestion as described below or proteins eluted for immunoblotting by addition of non-reduced laemmli buffer at 95°C for 10 min. The samples were analyzed by SDS-PAGE and western blot analysis using anti-HAX-1 and anti-FLAG and HRP-conjugated goat anti-rabbit IgG, Fc fragment specific (Jackson ImmunoResearch, 111-035-008).

### 4.10 On-beads digestion, mass spectrometry and protein identification

Following FLAG or Strep pulldown, beads were washed 3 times and tetraethylammonium bromide (TEAB) buffer was added. This was followed by on-bead protein reduction using 0.5 M tris (2-carboxyethyl) phosphine (TCEP) and alkylation of cysteines using Iodoacetamide (IAA; Sigma). The beads were digested overnight by trypsin (Trypsin Gold, Promega). After overnight digestion, peptides were recovered by filtration using filter plates (Millipore MultiScreen HTS DV Filter plate, 0.65 μm pore size) in 65% ACN/0.5% FA (acetonitrile/formic acid) solvent mixture. The tryptic digest was vacuum dried and redissolved in 0.5% FA/100% water and subjected to LC–MS/MS analysis.

LC–MS/MS analysis was performed on the Orbitrap Fusion Tribrid mass spectrometer coupled with U3000 RSLCnano UHPLC system (Thermo Fisher). Peptides were loaded on a PepMap C18 trap (100 μm i.d. x20 mm, 100 Å, 5 μm) for 10 min at 10 μl/min with 0.1% FA/H_2_O, then separated on a PepMap C18 column (75 μm i.d. x500 mm, 100 Å, 2 μm) for 70 min at 300 nl/min and a linear gradient of 5–45% ACN/0.1% FA. The Orbitrap Fusion was operated in the Top Speed mode at 3 s per cycle. The survey scans (m/z 375-1500) were acquired in the Orbitrap at a resolution of 120,000 at m/z 200 (AGC 4e5 with 50 ms maximum injection time). The multiply charged ions (2–7) with a minimal intensity of 1e4 counts were subject to MS/MS in HCD with a collision energy at 35% and an isolation width of 1.6 Th then detected in the orbitrap (AGC 1e5 with 105 ms maximum injection time). Dynamic exclusion width was set at ±7 ppm for 45 s.

Raw files were processed with Proteome Discoverer v. 2.3 (Thermo Fisher) and searches performed using SEQUEST search engine against the human Uniprot database (2019). The search parameters were trypsin digestion, two missed cleavages, 20 ppm mass tolerance for MS, 0.5 Da mass tolerance for MS/MS, with variable modifications of deamidation (NQ) and oxidation (M) and carbamidomethyl (C) as a static modification. Peptide false discovery rates (FDRs) were estimated based on matches to reversed sequences in a concatenated target-decoy database using Percolator and set at 0.01.

The mass spectrometry proteomics data has been deposited to the ProteomeXchange Consortium via the PRIDE partner repository with the data set identifier PXD023126.

### 4.11 In vitro ICC303 infection and cell detachment assay

HeLa cells were seeded at the concentration of 7.5 x 10^4^ cell/well in 24 well-plate 48 hr prior to infection. Bacteria were grown in LB overnight and 3 hr before infection, diluted 1:100 in DMEM (no IPTG was added in the primed bacteria). Before infection, the HeLa monolayer was washed three times with PBS and 0.5 ml of primed culture was added to each well with 20 μM z-VAD-fmk when required. The monolayers were incubated for 90 min, washed five times with PBS, then trypsinized and counted to quantify the number of remaining cells.

### 4.12 Statistical analysis

Data analysis was performed by GraphPad Prism software, using two-way ANOVA, and one-way ANOVA where specified, and Bonferroni post-test. Statistically significant was considered when *p* value is <.05.

## Supplementary Material

Additional supporting information may be found online in the Supporting Information section at the end of this article.

Figure S1

Figure S2

Figure S3

Supplementary Material

Supplementary Tables

## Data Availability

The data that support the findings of this study are openly available in ProteomeXchange Consortium via the PRIDE partner repository with the dataset identifier PXD023126.

## Take Away

- EspO homologues are found in EHEC, *Shigella, S. typhimurium* and some EPEC.
- EspO homologues interact with HAX-1.
- EspO protects infected cells from apoptosis.
- EspO joins a growing list of T3SS effectors that manipulate cell death pathways.
