# Supplementary figures and images for: The type III secretion system effector EspO of enterohaemorrhagic Escherichia coli inhibits apoptosis through an interaction with HAX-1
Source: Cell Microbiol. Author manuscript; Available in PMC 2022 Aug 13. (PMC7613270; doi:10.1111/cmi.13366)

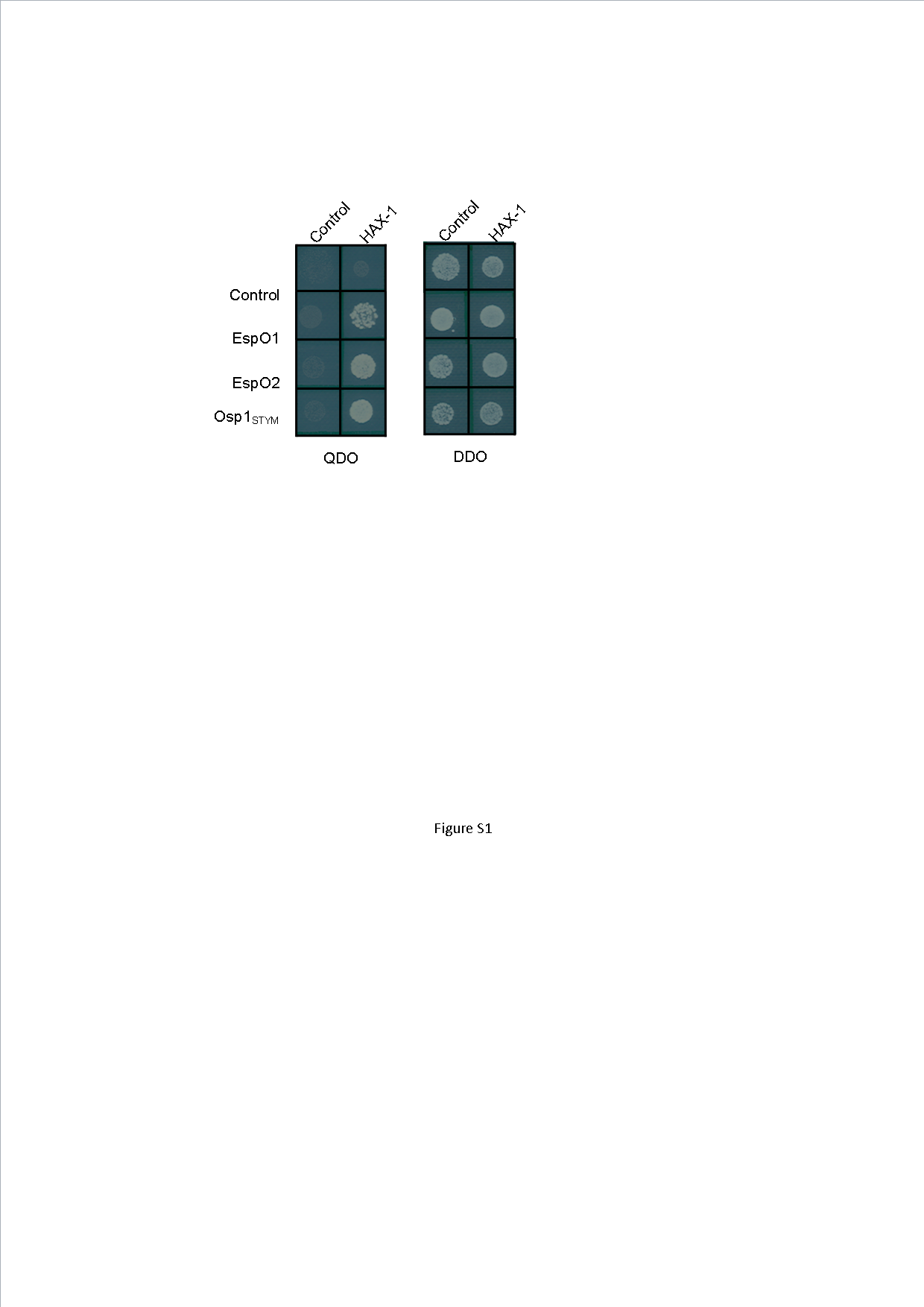

Supplement: Figure S1 [file EMS151439-supplement-Figure_S1.tif]

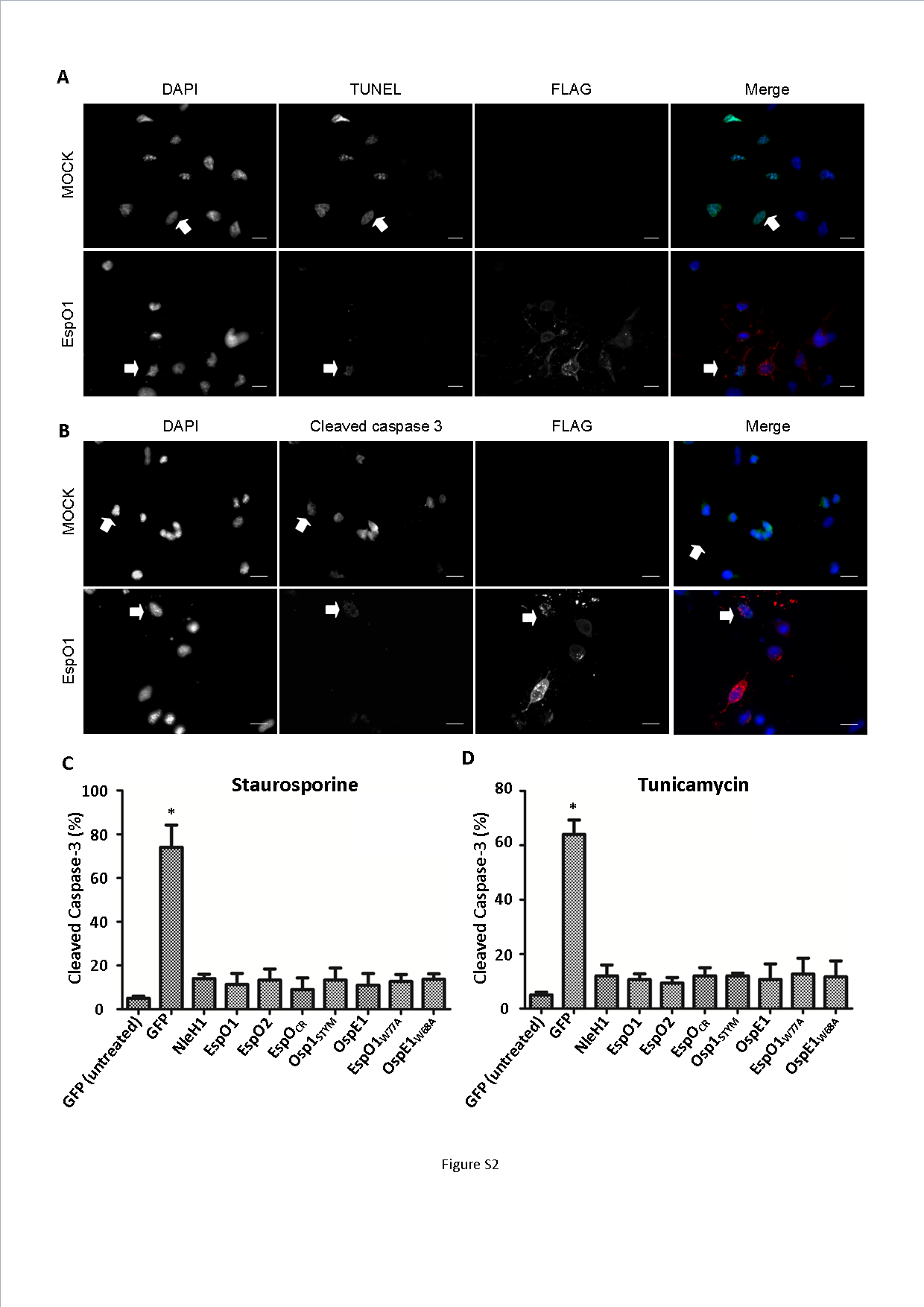

Supplement: Figure S2 [file EMS151439-supplement-Figure_S2.tif]

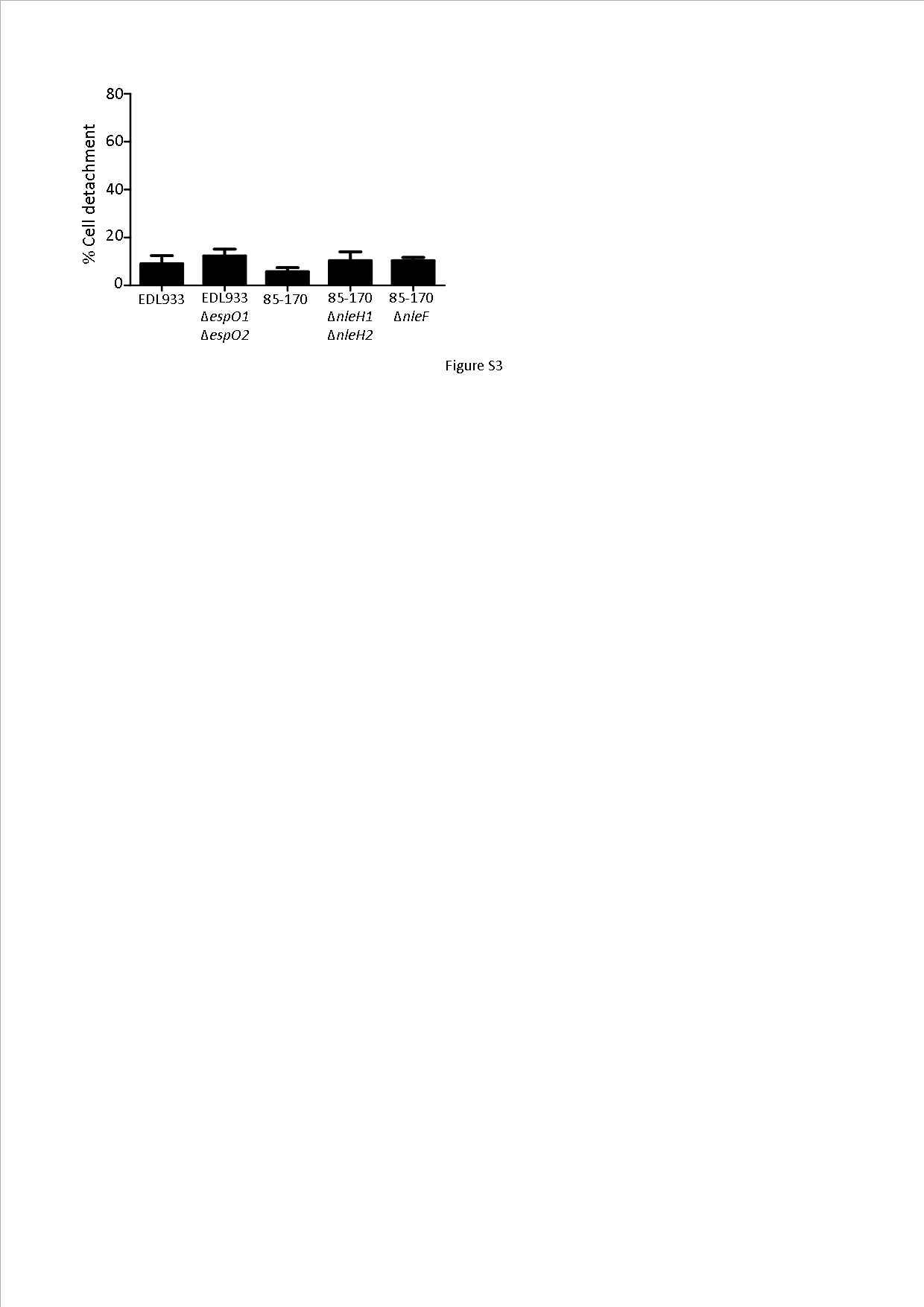

Supplement: Figure S3 [file EMS151439-supplement-Figure_S3.tif]
