# Supplementary material for: The type III secretion system effector EspO of enterohaemorrhagic Escherichia coli inhibits apoptosis through an interaction with HAX-1
Source: Cell Microbiol. Author manuscript; Available in PMC 2022 Aug 13. (PMC7613270; doi:10.1111/cmi.13366)
Supplement: Supplementary Material [file EMS151439-supplement-Supplementary_Material.docx]

**Supplementary Methods**

**Generation of EHEC *espO* and *nleF* deletion mutant**s

Gene deletion mutants were generated using the one-step PCR λ-red protocol {Datsenko, 2000 #15}. Disruption of *espO1* and *espO2* in EHEC EDL933 was performed with chloramphenicol and kanamycin resistance cassettes generated with primers C1/C2 and pKD3 or K1/K2 and pSB315 {Galán, 1992 #21} as templates, respectively. The PCR product of each resistance cassette contained 50 bp of the appropriate *espO* gene and was transformed into strains harbouring pKD46 (λ-Red recombinase expression plasmid). *nleF* was deleted from EHEC 85-170 (strain ICC1126) using primer pKD4F/R and the Kanamycin cassette from pKD4. First, primer pair 1F/R was used to amplify *nleF* with a 500 bp 5’ and 400 bp 3’ flanking sequence. The amplicon was cloned into TOPO Blunt II (Invitrogen) and *nleF* was subsequently removed by inverse PCR using primer pair 2F/R and replaced with the Kanamycin cassette. The linear product for recombination was amplified using primer pair 1F/R. Mutants were selected on LB plates containing the appropriate antibiotic and further confirmed by PCR.

***In vitro* EHEC infection assay**

48 h before infection, HeLa cells were seeded at the concentration of 7.5x104 cell/well in 24 well-plate. The monolayers were infected with primed bacterial cultures (MOI of 500:1) by centrifugation at 500g for 5 min. The cells were incubated for 2.5 h before washing 3 times by PBS. Then, 0.5 ml of fresh DMEM without supplement containing gentamicin 100µg/ml was added into each well. The monolayers were further incubated for 2.5 h before the cell detachment assay was carried as described previously.

**Table legend**

**Table S1. List of strains**

| Strains | Description | Source |
| --- | --- | --- |
| AH109 | Saccharomyces cerevisiae containing ADE2, HIS3, lacZ and MEI1 reporter genes, each of which depends on a GAL-4 responsive promoter | Clontech |
| E2348/69 | Wild type *E. coli* O127:H6 | {Levine, 1978 #82} |
| EPEC1 | *E. coli* O127:H6 E2348/69 ∆*map* ∆*espG* ∆*espF* ∆*espH* ∆IE5(*espG2,espC)* ∆IE6(*espL,nleB1,nleE1,efa/lifA)* ∆IE2(*espL*,nleB*,nleE2,efa/lifA-like)* ∆PP2(*nleF,cif*,espJ)* ∆PP3(*nleJ)* ∆PP4(*nleG,nleB,nleC,nleH*,nleD)* ∆PP6(*nleA/espI,nleH2,nleF,espO*)* ∆*espZ* | {Cepeda-Molero, 2017 #79} |
| ICC303 | *E. coli* O127:H6 E2348/69 ∆*nleH1*∆*nleH2*∆*nleF* | {Hemrajani, 2008 #26} |
| 85-170 | Spontaneous Nal^r^ derivative of *E. coli* O157:H7 85-170, stx- | {Stevens, 2004 #83} |
| ICC1126 | *E. coli* O157:H7 85-170 ∆*nleF* | This study |
| ICC232 | *E. coli* O157:H7 85-170 ∆*nleH1*∆*nleH2* | This study |
| EDL933 | Wild type *E. coli* O157:H7 stx- | {Miranda, 2004 #84} |
| ICC1182 | *E. coli* O157:H7 EDL933 ∆*espO1* | This study |
| ICC1183 | *E. coli* O157:H7 EDL933 ∆*espO2* | This study |
| ICC1184 | *E. coli* O157:H7 EDL933 ∆*espO1*∆*espO2* | This study |

**Table S2. List of plasmid**

| Plasmids | Description | Source |
| --- | --- | --- |
| pMAL-c2X | MBP tag expression vector | NEB |
| pICC2025 | pMAL::MBP derivative coding *espO1* | This study |
| pGBKT7 | yeast expression vector expressing protein fused with GAL4 DNA binding domain; Kan^r^ | Clontech |
| pICC1919 | pGBKT7 derivative encoding EHEC *espO1* | This study |
| pICC2019 | pGBKT7 derivative encoding EHEC *espO2* | This study |
| pICC1920 | pGBKT7 derivative encoding *C. rodentium espO* | This study |
| pICC1921 | pGBKT7 derivative encoding *S. flexneri* *ospE1* | This study |
| pICC2020 | pGBKT7 derivative encoding *espO1*_1-45_ | This study |
| pICC1929 | pGBKT7 derivative encoding *espO1*_46-91_ | This study |
| pICC2021 | pGBKT7 derivative encoding *espO1*_1-50_ | This study |
| pICC1930 | pGBKT7 derivative encoding *espO1_1-56_* | This study |
| pICC1922 | pGBKT7 derivative encoding *espO1*_W77A_ | This study |
| pGADT7 | yeast expression vector expressing protein fused with GAL4 DNA activation domain; Amp^r^ | Clontech |
| pICC1932 | pGADT7 derivative encoding *hax-1* | This study |
| pICC1925 | pGADT7 derivative encoding *hax-1*_1-65_ | This study |
| pICC1933 | pGADT7 derivative encoding *hax-1_1-100_* | This study |
| pICC2016 | pGADT7 derivative encoding *hax-1*_1-118_ | This study |
| pICC1934 | pGADT7 derivative encoding *hax-1*_65-279_ | This study |
| pICC1923 | pGADT7 derivative encoding *hax-1*_100-279_ | This study |
| pICC1928 | pGADT7 derivative encoding *hax-1*_118-279_ | This study |
| pICC1625 | pcDNA3.1 derivative for mammalian expression with TAP tag (3xFLAG-tag -TEV cleavage site – calmodulin binding peptide); Amp^r^ | {Young, 2014 #75} |
| pICC1927 | pcDNA-NTAP derivative encoding *espO1* | This study |
| pICC1924 | pcDNA-NTAP derivative encoding *espO1_W77A_* | This study |
| pICC2024 | pcDNA-NTAP derivative encoding *gfp* | {Young, 2014 #75} |
| pRK5-myc | mammalian expression plasmid, express N-terminal myc fusion protein; Amp^r^ | Addgene |
| pICC563 | pRK5-myc derivative encoding *gfp* | {Clements, 2011 #10} |
| pICC2018 | pRK5-myc derivative encoding *nleH1* | This study |
| pICC1931 | pRK5-myc derivative encoding EHEC *espO1* | This study |
| pICC2026 | pRK5-myc derivative encoding EHEC *espO2* | This study |
| pICC2027 | pRK5-myc derivative encoding *C. rodentium* *espO* | This study |
| pICC2028 | pRK5-myc derivative encoding *S.* Typhimurium *ospE1* | This study |
| pICC2029 | pRK5-myc derivative encoding *ospE1* (*S. flexnerie*) | This study |
| pICC2023 | pRK5-myc derivative encoding *espO1*_W77A_ | This study |
| pICC2022 | pRK5-myc derivative encoding *ospE1*_W68A_ | This study |
| pICC2481 | pRK5-myc derivative encoding *tccP* |  |
| pICC1661 | pRK5-myc derivative encoding *nleF* | {Pallett, 2014 #86} |
| pICC1926 | pCMV-myc derivative encoding *hax-1* | {Vafiadaki, 2009 #67} |
| pICC449 | pHM6-HA derivative encoding *nleH1* | {Hemrajani, 2008 #26} |
| pSA10 | pKK177-3 derivative encoding *lacI*; IPTG inducible; Amp^r^ | {Schlosser-Silverman, 2000 #57} |
| pICC1378 | pSA10 derivative encoding *espO1* | This study |
| pICC1376 | pSA10 derivative encoding *espO1*_W77A_ | This study |
| pICC2485 | pSA10 derivative encoding *espO1*_K49/50A_ | This study |
| pICC443 | pSA10 derivative encoding *nleH1* | {Hemrajani, 2008 #26} |
| pICC1769 | pACYC184 derivative encoding *nleF* | {Pallett, 2017 #85} |
| pICC2892 | pSA10 derivative encoding *espO1* with 3-x FLAG-TwinStrepII tag at C-terminus | This study |

**Table S3. List of primers**

| \| Name \| Sequence \| Restriction site \| \| --- \| --- \| --- \| \| FE1 \| AATGAATTCCCATTTTCAATCAAAAACAG \| EcoRI \| \| RE1 \| AATGGATCCCTATTCTTTTGTGTTGTGTATCTC \| BamHI \| \| RE2 \| AATGGATCCCTATGTATAGCTATCTACATTAC \| BamHI \| \| FE2 \| ATAGAATTCGTGTACCAAAAAAAAGCCTGTAGTTTTG \| EcoRI \| \| RE3 \| AATGGATCCCTATTTTTTTTGGTACACTGTATAG \| BamHI \| \| RE4 \| ATAGGATCCCTACATGTCAAAACTACAGGC \| BamHI \| \| FE6 \| atagaattcCCATTTTCAATCAAAAACAGATTTTC \| EcoRI \| \| RE6 \| atactcgagCTATTCTTTTGTGTTGTGTATCTC \| XhoI \| \| FE7 \| GCTGAATTCATGCCATTTTCAATCAAAAAC \| EcoRI \| \| RE7 \| GCTCTGCAGTTCTTTTGTGTTGTGTATC \| PstI \| \| FE8 \| GCTGAATTCCCATTTTCAATCAAAAACAGATTTTC \| EcoRI \| \| RE8 \| GCTGGATCCCTATTCTTTTGTGTTGTGTATC \| BamHI \| \| FE9 \| GCTGGATCCATGCCATTTTCAATCAAAAAC \| BamHI \| \| RE9 \| GCTGAATTCTATTCTTTTGTGTTGTGTATC \| EcoRI \| \| F2 \| ATAGAATTCCCATTTTCAATAAAAAGTATTTTTTC \| EcoRI \| \| R2 \| ATAGGATCCCTATGATTTGTTTGTATTATTTG \| BamHI \| \| F3 \| GCTGGATCCATGCCATTTTCAATAAAAAG \| BamHI \| \| R3 \| GCTGAATTCTATGATTTGTTTGTATTATTTG \| EcoRI \| \| FC1 \| GCTGAATTCCCATTGTCAATAAGAAATATATTTTC \| EcoRI \| \| RC1 \| GCTGGATCCTCAGGATTTATTTGAGTTATTAA \| BamHI \| \| FC2 \| GCTGGATCCGGAGCACCATTGTCAATAAGAAATATAT \| BamHI \| \| RC2 \| GCTGAATTCTCAGGATTTATTTGAGTTATTAATCTCG \| EcoRI \| \| FO1 \| GCTGAATTCCTTACACAAACTATATTCCCTTGTC \| EcoRI \| \| RO1 \| GCTGGATCCTCAGAAATCAGAATACCGTTG \| BamHI \| \| FO2 \| GCTGGATCCGGAGCACTTACACAAACTATATTCCC \| BamHI \| \| RO2 \| GCTGAATTCTCAGAAATCAGAATACCGTTGGCTTT \| EcoRI \| \| FT1 \| GCTGGATCCGGAGCACCATTTTCAATCAAAAAC \| BamHI \| \| RT1 \| GCTGAATTCTCATTCTTTTGTGTTGC \| EcoRI \| \| FN1 \| tgggatcCGCGCTATCACCATCTTCTG \| BamHI \| \| RN1 \| GGCCAagctTCTAAATTTTACTTAATACCAC \| HindIII \| \| 68AF \| GCTACAGAAATGCAAGCGTTGACAAAGATAAATG \| - \| \| 68AR \| CATTTATCTTTGTCAACGCTTGCATTTCTGTAGC \| - \| \| 77AF \| GTTACTGAGATGACAGCGCTATCTAAAACTATAG \| - \| \| 77AR \| CTATAGTTTTAGATAGCGCTGTCATCTCAGTAAC \| - \| \| HF1 \| ATAGAATTCAGCCTCTTTGATCTCTTCCGGGG \| EcoRI \| \| HF2 \| ATAGAATTCTTCAGCTTCAGCCCAGGAGGAGGG \| EcoRI \| \| HF3 \| ATAGAATTCCCTTCCCATCCTCCTGAACTTCCAGG \| EcoRI \| \| HF4 \| ATAGAATTCAGACTACGGGAGGGACAGACACTTCGGG \| EcoRI \| \| HR1 \| ATAGGATCCCTACCGGGACCGGAACCAACGTC \| BamHI \| \| HR2 \| ATAGGATCCCTAAAGCCGAAGCCAAATTCCTCAG \| BamHI \| \| HR3 \| ATAGGATCCCTAAGGTCCAGGCCCCCATATC \| BamHI \| \| HR4 \| ATAGGATCCCTAAGCATTGAGTCCCGAAGTG \| BamHI \| \| C1 \| CAACTTATAACGGAATAGCATAAAAACACTTTTCATGGAGCAAAGGAGAAAACATGTGTAGGCTGGAGCTGCT \| - \| \| C2 \| GTCAGGTGTTTCGGGTCTGTGGCTTTTTGTGTTCTCTGGTAAGTGGTTGCATATGAATATCCTCCTTAGTTCC \| - \| \| K1 \| CTAAATACATTAAATTGCACTATAAGAGAAAGACATAATGTGAGGATAAAATGTGTAGGCTGGAGCTGCTTCG \| - \| \| K2 \| GTTAGGAAGTTATTCATGGCTAGTGTACCAACTCTTTTCTGGGCTAATAGCATATGAATATCCTCCTTAGTTCC \| - \| \| KK1 \| CTATACAGTGTACCAAGCAGCAGCCTGTAGTTTTG \| - \| \| KK2 \| CAAAACTACAGGCTGCTGCTTGGTACACTGTATAG \| - \| \| 1F \| GATATGGAAGATACAACAAAAGTGTT \| - \| \| 1R \| ACAGAAGAGGCAGGCCAGCA \| - \| \| 2F \| TTAGAGGCCTGTGAGCCTGT \| - \| \| 2R \| ATCAAAACCCCCTTAACAAATAAATC \| - \| \| pKD4F \| TGTGTAGGCTGGAGCTGCTTC \| - \| \| pKD4R \| CATATGAATATCCTCCTTAGTTCC \| - \| \| 3F \| GTAAAGGTGGTAATGCTGTCGT \| - \| \| 3R \| GAAGGAATACAAAGCCGTCAC \| - \| |
| --- | --- | --- | --- | --- | --- | --- | --- | --- | --- | --- | --- | --- | --- | --- | --- | --- | --- | --- | --- | --- | --- | --- | --- | --- | --- | --- | --- | --- | --- | --- | --- | --- | --- | --- | --- | --- | --- | --- | --- | --- | --- | --- | --- | --- | --- | --- | --- | --- | --- | --- | --- | --- | --- | --- | --- | --- | --- | --- | --- | --- | --- | --- | --- | --- | --- | --- | --- | --- | --- | --- | --- | --- | --- | --- | --- | --- | --- | --- | --- | --- | --- | --- | --- | --- | --- | --- | --- | --- | --- | --- | --- | --- | --- | --- | --- | --- | --- | --- | --- | --- | --- | --- | --- | --- | --- | --- | --- | --- | --- | --- | --- | --- | --- | --- | --- | --- | --- | --- | --- | --- | --- | --- | --- | --- | --- | --- | --- | --- | --- | --- | --- | --- | --- | --- | --- | --- | --- | --- | --- | --- | --- | --- | --- | --- | --- | --- | --- | --- | --- | --- | --- | --- | --- | --- | --- | --- | --- | --- | --- | --- | --- | --- | --- | --- | --- | --- | --- | --- | --- | --- | --- |
|  |

Supplementary tables: S4-S8

S4: Common proteins identified in EPEC-1 EspO1 FLAG and EPEC-1 EspO1 Strep pulldowns in HT-29 infected cells. S5-S9: Proteins identified from EPEC-1EV FLAG (S5), EPEC-1 EV STREP (S6), EPEC-1 EspO1 FLAG (S7) and EPEC-1 EspO1 STREP (S8) pulldowns in HT-29 infected cells.

**Fig. S1. OspE1_STYM_ binds HAX-1**.

Yeast AH109 co-transformed with pGBKT7- *OspE1_STYM_* and pGADT7-HAX-1 grew on selective medium (QDO), whereas yeast co-transformed with pGBKT7- *OspE1_STYM_* and empty pGADT7 did not. Growth on non-selective media (DDO) indicated a successful plasmid co-transformation.

**Figure S2. EspO homologues inhibit TUN and STS-induced apoptosis.**

(A) and (B) Representative images of cells expressing EspO(FLAG) or Mock transfected, treated with STS, and apoptosis visualized by DNA fragmentation (fluoroscein-12-dUTP) (**A**) or cleaved caspase-3 (B). (C) and (D) Cleaved caspase-3 was quantified in cells expressing GFP, EspO1, EspO2, EspO_CR_, OspE1_STYM_, OspE1, EspO_W77A_ and OspE1_W68A_ and treated with STS (**C**) or TUN (**D**). Anti-Myc, DAPI and anti–cleaved caspase-3 (green) were used to detect by immunofluorescence FLAG-EspO1 and EspO1_W77A_, myc-NleH1, DNA, or active caspase-3 respectively. Ectopic expression of EspO1, EspO1_W77A_ or NleH1 or zVAD treatment prevented cleavage of procaspase-3 induced by STS or TUN compared with GFP-transfected cells. Significance (P < 0.05) was tested using 2 way ANOVA (Bonferroni test). Results are the average of 3 independent biological repetitions.

**Figure S3. Cell detachment induced by EHEC**

Quantification of cell detachment following infection of HeLa cells with EHEC EDL933, EDL933 ∆*espO1*∆*espO1*, EHEC 85-170, 85-170 ∆*nleH1*∆*nleH2*, and 85-170 ∆*nleF*. No significant difference in cell detachment was observed between strains. The results were obtained from 3 independent biological repeats.
